# Does procalcitonin have clinical utility in the management of paediatric community-acquired pneumonia? A PRO/CON debate

**DOI:** 10.1093/jacamr/dlab153

**Published:** 2021-10-22

**Authors:** Kathleen Chiotos, Jeffrey S Gerber

**Affiliations:** 1 Department of Anesthesiology and Critical Care, Children’s Hospital of Philadelphia, Philadelphia, PA, USA; 2 Department of Pediatrics, Children’s Hospital of Philadelphia, Philadelphia, PA, USA; 3 Center for Pediatric Clinical Effectiveness, Children’s Hospital of Philadelphia, Philadelphia, PA, USA

## Abstract

Although the overwhelming majority of community-acquired pneumonia (CAP) in children is caused by viral infections, treatment of CAP is among the most common indications for antibiotic use in children. This is largely driven by the imprecision of clinical diagnostic tools to differentiate viral from bacterial pneumonia and highlights the need for improved approaches to optimizing management of CAP in children. In this issue of *JAC-Antimicrobial Resistance*, we present a PRO/CON debate that discusses the clinical utility of procalcitonin in children with CAP.

Community-acquired pneumonia (CAP) accounts for approximately 1.5 million ambulatory visits per year, and is the third most prevalent condition and most common indication for antibiotics among paediatric inpatients.^1–4^ While the overwhelming majority of children hospitalized with CAP in the USA recover uneventfully, 7% require mechanical ventilation or vasopressors or die—and globally, CAP accounts for 15% of deaths in children under 5 years old.^5^^,^^6^ Multiple studies suggest that the majority of paediatric CAP in the post-pneumococcal vaccine era is caused by viruses: for example, in the USA, viruses alone were identified in 66% of children hospitalized with CAP, while bacterial aetiologies were detected in just 15%.^7^ Similar findings were demonstrated in children presenting to primary care clinics in Malawi, where 91% of children had a virus identified.^8^ Despite the predominance of viral aetiologies, approximately 70% of children diagnosed with CAP are treated with antibiotics.^9^^,^^10^ Further, there is significant hospital- and practice-level variation in antibiotic use for CAP across settings.^3^^,^^11^ Given the imprecision of currently available diagnostic tests to differentiate bacterial versus viral aetiologies of pneumonia this is not surprising, but nevertheless highlights the need for improved diagnostic tools to identify the small number of children with CAP who benefit from antibiotics, while reducing unnecessary antibiotic exposure in the large number who do not. In this PRO/CON debate, we consider the question: does procalcitonin have clinical utility in the management of children with CAP?

Procalcitonin is primarily produced by C cells in the thyroid gland as a precursor of the hormone calcitonin under normal physiological conditions. However, in the presence of a bacterial infection, proinflammatory cytokines, including TNF-α, IL-1β and IL-6, upregulate gene expression of the *CALC-1* gene, which drives production of procalcitonin in multiple cells throughout the body, leading to a rapid rise in the blood procalcitonin level. As the bacterial infection is controlled with adequate antibiotic therapy, procalcitonin is expected to decrease by 50% every 1–2 days. Cytokines more typically produced in the setting of a viral infection, such as IFNγ, downregulate *CALC-1* expression, contributing to preferential rises in procalcitonin in response to bacterial infections. While these characteristics make procalcitonin an appealing biomarker to diagnose bacterial infections, as well as to assess response to antibiotic therapy, it is imperfect. For example, non-bacterial infections, including malaria, as well as non-infectious conditions, such as malignancies, surgery, bowel ischaemia and burns, can also lead to elevations in elevations in procalcitonin.^12^

Indeed, the performance characteristics of procalcitonin for diagnosis of bacterial pneumonia in adults are marginal, with no identified threshold that distinguishes viral from bacterial aetiologies with sufficient accuracy to withhold antibiotic initiation.^13^^,^^14^ Few studies have evaluated the performance characteristics of procalcitonin in paediatric CAP, but the CDC Etiology of Pneumonia in the Community (EPIC) study identified a procalcitonin cut-off of <0.25 ng/mL as 85% sensitive and 45% specific for identifying children without bacterial pneumonia, with a negative predictive value of 96%. A troublesome finding of this study was the wide distribution of procalcitonin values among children with exclusively viral infections; for example, 64% of children with a procalcitonin value ≥0.5 ng/mL, a threshold used to identify likely bacterial pneumonia in adult trials, had only a viral infection identified. This raises questions as to whether an over-reliance on procalcitonin could have an unintended consequence of increasing antibiotic use in children. Further, while no children with a procalcitonin value of <0.1 ng/mL had a bacterial aetiology identified, it is unclear if and how individual antibiotic prescribing behaviour would actually change in response to these data—that is, would having a low procalcitonin value in hand sway a provider away from antibiotic treatment?^15^

Over two dozen randomized controlled trials (RCTs) have considered this question in adults, examining the impact of procalcitonin-guided algorithms for antibiotic use in adults with acute lower respiratory tract infection (LRTI) in emergency department (ED ), primary care and ICU settings. Most of these studies demonstrated reductions in antibiotic initiation as well as antibiotic duration, though notably, a trial conducted in 14 EDs in the USA with high adherence to the Joint Commission core measures for management of CAP demonstrated that a procalcitonin-based guideline for antibiotic use had no impact on total antibiotic exposure or rate of antibiotic use.^16^^,^^17^ Two small paediatric RCTs have compared a procalcitonin-guided algorithm with standard care, and both demonstrated reductions in total length of antibiotic treatment in the procalcitonin-guided group to approximately 5 days.^18^^,^^19^ Subsequently, however, multiple paediatric RCTs and observational studies have supported shorter durations of 5 days of antibiotic therapy among children with uncomplicated CAP, such that if these paediatric procalcitonin trials were repeated today with this new evidence-based standard implemented in the non-procalcitonin group, the difference in antibiotic duration between groups may disappear.^20^^,^^21^ Collectively these data raise important questions related to the generalizability of trial data to real-world paediatric practice, as the results are influenced by variability in baseline antibiotic prescribing practices, co-interventions, including antibiotic stewardship, and local implementation of an ever-evolving evidence base.

In this issue of *JAC-Antimicrobial Resistance*, Florin and Williams argue in favour of procalcitonin use, with their ‘pro’ position resting on the value of procalcitonin in excluding bacterial pneumonia at very low values (i.e. <0.1 ng/mL), which could substantially reduce antibiotic use in children where viral infections predominate.^22^ Banerjee argues the ‘con’ position, focusing primarily on the lack of paediatric data establishing appropriate procalcitonin thresholds and conflicting findings in adult trials evaluating the utility of procalcitonin-guided algorithms.^23^ As readers consider these two viewpoints, we encourage reflection on if, and how, procalcitonin may improve upon current, evidence-based management of paediatric CAP; how diagnostic test stewardship applied to procalcitonin use may improve its utility; and how future studies should be designed to answer these questions.

## Funding

This work was supported by the Agency for Healthcare Research and Quality [K12-HS026393 to K.C.]. The funder did not participate in this work.

## Transparency declarations

None to declare.

### Author contributions

K.C. and J.S.G. conceived of the content included in this manuscript. K.C. drafted the initial manuscript and J.S.G. critically reviewed the manuscript.
